# Serum Golgi protein 73 levels and liver pathological grading in cases of chronic hepatitis B

**DOI:** 10.3892/mmr.2014.3114

**Published:** 2014-12-18

**Authors:** ZHENGJU XU, XINGNAN PAN, KAIPENG WEI, HONGBING DING, MEIJUAN WEI, HUANWEN YANG, QIAN LIU

**Affiliations:** 1 Clinical Liver Center, The 180th Hospital of the People’s Liberation Army, Quanzhou, Fujian 362000, P.R. China; 2 Central Laboratory of Clinical Hepatology, The 180th Hospital of the People’s Liberation Army, Quanzhou, Fujian 362000, P.R. China; 3 Liver Center Clinical Pathology, The 180th Hospital of the People’s Liberation Army, Quanzhou, Fujian 362000, P.R. China

**Keywords:** Golgi protein 73, hepatitis B virus, hepatic inflammation, liver fibrosis, immunohistochemistry

## Abstract

The present study was designed to assess the correlation between serum Golgi protein 73 (GP73) and liver pathological grading and staging in patients with chronic hepatitis B (CHB). Two hundred and fifty-three patients with chronic hepatitis B virus (HBV) infections were enrolled in the present study. All patients received a serum GP73 test, and 91 CHB patients underwent liver biopsy. GP73 expression in liver tissue was assessed by immunohistochemical analysis. The results indicated that serum GP73 levels were positively correlated with disease progression in patients with chronic HBV infection (*r*=0.677). There was no significant difference in serum GP73 levels between hepatitis B e antigen-positive and −negative patients (P>0.05). There were also no significant differences in serum GP73 levels among specimens with varying HBV DNA contents (P>0.05). Serum GP73 levels were positively correlated with increased liver pathological grading (*r*=0.737) and staging (*r*=0.692), and immunohistochemical analysis indicated that GP73 protein expression increased concurrently with liver pathological grading and staging. In conclusion, serum GP73 was found to be correlated with liver pathological grading and staging in patients with CHB, and may be an effective indicator for the evaluation of disease progression. However, serum GP73 levels were not associated with HBV replication.

## Introduction

Hepatitis B virus (HBV) is a highly infectious virus with an estimated two billion people infected worldwide (1). Approximately 240 million individuals worldwide are chronically infected with HBV, leading to ~600,000 mortalities as a result of acute or chronic hepatitis B annually (2). One of the major risk factors of HBV is acute or chronic liver disease which may progress into HBV-associated liver failure, leading to an increased risk of mortality from liver cirrhosis or primary hepatocellular carcinoma (3). Chronic HBV infection may result in sustained hepatic injury and liver fibrosis; therefore, an accurate method for the assessment of hepatic injury and liver fibrosis is vital for patients with chronic HBV infections. At present, a lack of accurate, reproducible and easily applied assessments for hepatic injury and liver fibrosis in patients with HBV is a major limitation of the clinical management of HBV. Current clinical indicators, including ultrasonography, liver function tests, markers of coagulation function and serum markers for liver fibrosis may reflect disease progression in patients with chronic hepatitis B (CHB) (4). Liver biopsies provide an accurate reflection of the progression of liver disease in patients with CHB (1). However, the clinical applications of this test remain limited due to its invasive nature. Therefore, the identification of a simple, non-invasive serum marker for evaluating disease progression in patients with CHB is required.

Golgi protein 73 (GP73), a novel transmembrane protein, is expressed in human epithelial cells (5). In normal human liver tissue, GP73 is primarily expressed in biliary epithelial cells and rarely expressed in healthy hepatocytes (6,7). Studies have revealed that hepatocellular GP73 mRNA levels and protein expression are significantly upregulated in acute and chronic hepatitis, regardless of the etiology, and accompanies the advanced fibrogenesis stage (7.8). Furthermore, a significant increase in GP73 protein expression has been reported in the liver tissue of patients with CHB and GP73 was highly expressed in liver tissue infected with HBV and adenovirus (9,10). Multiple studies have demonstrated that increased serum GP73 protein concentrations were positively correlated with the progression of chronic liver disease (8,11). However, the correlation between serum GP73 protein levels and liver pathological grading or fibrosis staging in patients with CHB has remained unclear, and the association between serum GP73 protein levels and HBV replication are under dispute (12,13). The current study aimed to evaluate the correlation between serum GP73 protein levels and liver pathological grading and staging in patients with CHB. Serum GP73 levels were also investigated in order to determine the correlation between serum GP73 protein levels and HBV replication.

## Materials and methods

### Subjects

A total of 253 patients with chronic HBV infections were enrolled in the present study, including 183 patients with CHB, 35 patients with acute-on-chronic liver failure (ACLF) and 35 patients with HBV-associated decompensated liver cirrhosis (HBV-DLC). Patients with chronic HBV infections enrolled in this study were admitted to the 180th Hospital of the People’s Liberation Army (Quanzhou, China) during the period between January 2012 and September 2013. The demographic and clinical data of each patient were recorded. Thirty healthy individuals were selected as normal control subjects during the same period. The study was approved by the Ethical Review Committee of the 180th Hospital of People’s Liberation Army. All participants provided written informed consent.

The diagnoses of CHB and HBV-DLC were made according to the program of prevention and cure for viral hepatitis (14). Briefly, cases of CHB were classified as mild, moderate and severe. A diagnostic classification of mild CHB was defined as albumin (ALB) levels ≥35 g/l, alanine aminotransferase (ALT) levels ≤3 upper limits of normal (ULN), total bilirubin (TBIL) levels ≤2 ULN and prothrombin activity (PTA) >70%. A diagnostic classification of moderate CHB required criterion of ALB 32–35 g/l, ALT >3 ULN, TBIL 2–5 ULN and a PTA of 60–70%. The diagnostic criterion for severe CHB required an ALB ≤32 g/l, ALT >3 ULN, TBIL >5 ULN and a PTA of 40–60%. HBV-DLC diagnostic criteria were advanced liver cirrhosis and a Child-Pugh score (15) of B or C class. The diagnosis of ACLF was made according to the consensus on ACLF recommended by the Asian Pacific Association for the study of the liver (16). Patients with liver damage caused by hepatitis A, C, D and E or other factors were excluded. The serum GP73 level, hepatitis B e antigen (HBeAg), HBV DNA and liver function of each patient were measured. Prior to the initiation of drug therapy and following six months of treatment, serum samples were collected and stored at −80°C for subsequent investigations.

### Determination of serum GP73 level

Quantitative determination of GP73 in serum was performed using a commercially available enzyme-linked immunosorbent assay (ELISA) kit according to the manufacturer’s instructions (Beijing Hotgen Biotech Co., Ltd, Beijing, China). All reagents were provided in the kit. Briefly, a 20 μl serum blood sample was added to each well of the ELISA microplate with 50 μl dilution solution, and the microplate was sealed and incubated at 37°C for 60 min. Subsequently, 100 μl ELISA reagent was added to each well and the microplate was sealed and incubated at 37°C for 30 min. All solutions were removed, and each well was washed with phosphate-buffered saline (PBS)/Tween (Fuzhou Maixin Biotechnology Development Co., Ltd, Fuzhou, China)five times. Chromogenic substrates A and B (50 μl) were added to each well and the plate was incubated in darkness at 37°C for 15 min. Subsequently, 50 μl stop solution was added to each well, and the plate was read in a Bio-Rad 860 microplate reader (Bio-Rad Laboratories, Inc., Hercules, CA, USA).

### Determination of serum liver fibrosis indices

Serum liver fibrosis indices, including hyaluronic acid (HA), type IV collagen (CIV), laminin (LN) and type III procollagen (PIIINP), were determined using the up-converting luminescence technique with an up-converting phosphor bioanalytical system (Beijing Hotgen Biotech Co., Ltd.) according to the manufacturer’s instructions.

### Liver pathology and immunohistochemistry

#### Liver pathology

Liver biopsies were obtained from 91 CHB patients using 16 G disposable needles (C. R. Bard, Inc., Murray Hill, NJ, USA). The liver biopsy specimens were considered reliable when the liver specimen length was ≥1.5 cm or the portal tract number was ≥6. The liver biopsy specimens were fixed in 4% formaldehyde (Shanghai Xi Hua Trade Co., Ltd, Shanghai, China), embedded in paraffin (Maoming Huayue Group Co., Ltd, Maoming, China) and cut into 4-μm sections, and conventionally stained with hematoxylin and eosin (Shanghai Xi Hua Trade Co. Ltd), reticular fiber stain and Masson’s trichrome (Beijing Sequoia Jinqiao Biological Technology Co. Ltd, Beijing, China). Images were acquired using an Olympus BX51 microscope (Tokyo, Japan). According to the Scheuer scoring system (17), hepatic inflammation activity grade (G) was divided into G0 (no hepatic necroinflammation) and G1–G4, and liver fibrosis stage (S) was divided into S0 (no fibrosis) and S1–S4. Liver specimens were interpreted by an experienced liver pathologist.

#### Immunohistochemistry

Immunohistochemical (IHC) staining of liver sections was performed using the EliVision™ Plus IHC kit (Fuzhou Maixin Biotechnology Development Co., Ltd) according to the manufacturer’s instructions. All reagents were provided in the kit. Briefly, the tissue microarray blocks were cut into 3-μm sections, deparaffinized with xylene and rehydrated through a graded alcohol series. Antigen retrieval was performed using a high-temperature and high-pressure antigen repairing method. Following rinsing in Tris-buffered saline (pH 7.6), the sections were immersed in 3% H_2_O_2_ to block endogenous peroxidase activity. Samples were subsequently incubated with GP73 mouse monoclonal antibody (Beijing Hotgen Biotech Co., Ltd.) overnight at 4°C. Following washing with Tris-buffered saline, GP73 horseradish peroxidase-labeled goat anti-mouse antibody (Fuzhou Maixin Biotechnology Development Co., Ltd) was added to each section for 30 min at 37°C, and then visualized using 3,3′-diaminobenzidine (Beijing Sequoia Jinqiao Biological Technology Co., Ltd.). Counterstaining was performed using hematoxylin. PBS in place of the primary antibody was used as a blank control. According to the staining intensity, based on the semi-quantitative evaluation (6) of GP73 expression in liver tissue, GP37 staining was divided into five categories: negative (no expression), weakly positive (fine brown particle), positive (coarse brown particle), moderately positive (lumpy brown particle) and highly positive (chunky dark brown particle).

#### Determination of serum HBV DNA content

The serum HBV DNA was determined by the fluorescent quantitative polymerase chain reaction (qPCR) technique according to the manufacturer’s instructions (Shanghai Fosun Industrial Limited by Share, Ltd, Shanghai, China). Primer sequences were synthesized by Shanghai Shenyou Biotechnology Co., Ltd and fluorescence was measured using the LightCycler 600 real-time fluorescence quantitative PCR instrument manufactured by Roche (Roche Diagnostics, Basel, Switzerland). The sensitivity of HBV DNA for the determination was <5.0E+02 copies/ml.

#### Biochemical indices of liver function analysis

The liver function-associated biochemical indices of ALB, TBIL, ALT and aspartate aminotransferase (AST) were determined by the chemical colorimetric method with a TBA-120 FR fully automatic biochemical analyzer (Toshiba, Tokyo, Japan) according to the manufacturer’s instructions (Beijing Condor-Teco Medical Technology Co., Ltd, Beijing, China).

#### Changes in serum GP73 following antiviral treatment

A total of 86 patients with CHB received antiviral therapy [0.5 mg Entecavir (Zhengda Tianqing Pharmaceutical Group Ltd, Jiangsu, China) every morning prior to eating ordrinking, administered orally, once a day over one year; or 5×10^6^ units recombinant human interferon α1b (Kexing Biological Engineering Co., Ltd, Shenzhen, China), administered by subcutaneous injection, once every other day over a course of six months)]. Changes in condition were observed and the serum levels of GP73 were detected prior to the initiation of drug therapy and following six months of treatment. Prognoses were graded as: 1, Clinical remission, where clinical symptoms had disappeared and liver function had returned to normal or 2, clinical disease progression, where clinical symptoms were not relieved and were accompanied by recurrent or persistent abnormal liver function.

#### Statistical analysis

All values are expressed as the mean ± standard deviation, and all statistical analyses were performed using GraphPad Prism version 5.0 (Graphpad Software Inc., La Jolla, CA, USA). The difference of the means of the data that satisfied the homogeneity of variance were tested with analysis of variance (ANOVA), and comparison of the data that did not satisfy the homogeneity of variance was analyzed with ANOVA following rank transformation. The correlation analyses among variables were performed using Pearson’s correlation analysis and linear regression analysis. P<0.05 was considered to indicate a statistically significant difference between values.

## Results

### General characteristics

Between January 2012 and September 2013, 253 patients with chronic HBV infections were enrolled in the present study, including 213 males and 40 females, aged 20–65 years, with a mean age of 39.75±14.69 years. The subjects included 183 patients with CHB, including 53 with mild CHB, 80 with moderate CHB and 50 with severe CHB, as well as 35 patients with ACLF and 35 patients with HBV-DLC. The serum GP73 levels, HBeAg, HBV DNA and liver function of each patient were assessed (Table I).

### Serum GP73 levels are positively correlated with HBV clinical disease progression

**Figure 1:**
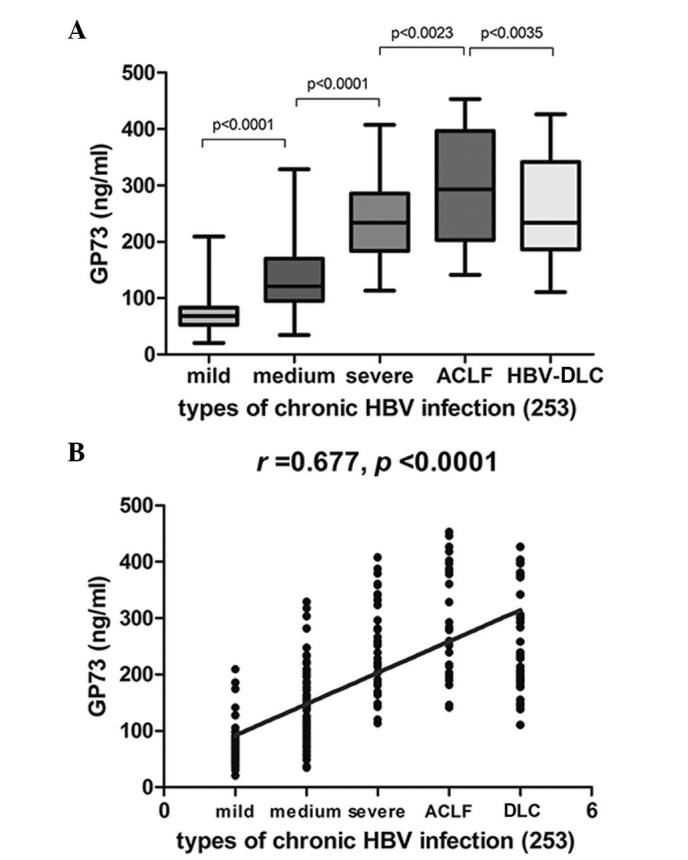
Correlation between serum GP73 level and disease progression in patients with chronic HBV infection. (A) Serum GP73 levels in patients with various types of chronic HBV infection. (B) Correlation between serum GP73 levels and clinical outcomes of patients with chronic HBV infections. HBV, hepatitis B virus; GP73, Golgi protein 73; ACLF, acute-on-chronic liver failure; HBV-DLC, HBV-associated decompensated liver cirrhosis.

Elevation of serum GP73 was observed in patients with mild, moderate and severe CHB, patients with HBV-DLC and patients with hepatitis B-associated ACLF (Fig. 1A). Serum GP73 levels were positively correlated with disease progression in patients with chronic HBV infections (Fig. 1B; *r*=0.677; P<0.0001).

### Serum GP73 level is not correlated with HBV replication

Serum HBeAg and HBV DNA are two major indices of HBV replication. The association between serum GP73 levels and HBV replication was investigated in the present study. The subjects included 183 patients with CHB, including 117 (63.93%) with HBeAg-positive CHB and 66 (36.07%) with HBeAg-negative CHB. Serum levels of GP73 were not significantly different between patients with HBeAg-positive CHB and those of patients with HBeAg-negative CHB (*t*=0.212; P>0.05).

According to the serum HBV DNA content, 183 patients with CHB were divided into four groups: <1.0E+06, 1.0E+06 – 1.0E+07, 1.0E+07 – 1.0E+08 and >1.0E+08 copies/ml. The association between serum GP73 levels and HBV DNA contents are exhibited in Table I. There was no significant difference in serum GP73 levels between the four groups (*F*=0.513; P>0.05).

### Serum GP73 levels are correlated with liver function-associated biochemical indices

**Figure 2:**
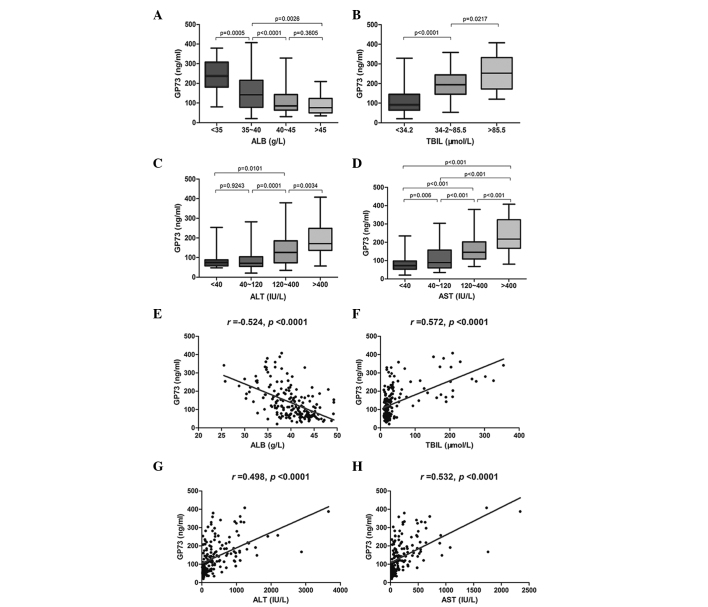
Serum GP73 level closely correlates with serum ALB, TBIL, ALT and AST levels. Serum GP73 levels in patients with various (A) ALB, (B) TBIL, (C) ALT and (D) AST levels. Correlations between serum GP73 level and (E) ALB, (F) TBIL, (G) ALT and (H) AST levels. GP73, Golgi protein 73; ALB, albumin; TBIL, total bilirubin; ALT, alanine aminotransferase; AST, aspartate aminotransferase.

Serum ALT and AST levels are the most commonly used indicators of liver cell injury. In addition, serum TBIL levels are associated with hepatic necrosis, whilst serum ALB levels reflect hepatic synthetic function. These four serological biochemical indices are frequently used to evaluate the degree of hepatic injury and necrosis (18). The correlation between GP73 and liver function-associated biochemical indices was therefore evaluated. The serum ALB, TBIL, ALT and AST levels of 183 patients with CHB were determined. The patients were divided into four groups according to serum ALB levels (<35 g/l, 24 cases; 35–40 g/l, 61 cases; 40–45 g/l, 78 cases and >45 g/l, 20 cases) and three groups according to serum TBIL levels (≤34.2 μmol/l, 139 cases; 34.2–85.5 μmol/l, 20 cases and ≥85.5 μmol/l, 24 cases). Patients were also divided into four groups according to serum ALT levels (<40 IU/l, 18 cases; 40–120 IU/l, 52 cases; 120–400 IU/l, 59 cases and >400 IU/l, 54 cases), and four groups according to serum AST levels (<40 IU/l, 38 cases; 40–120 IU/l, 65 cases; 120–400 IU/l, 53 cases and >400 IU/l, 27 cases). The associations between serum GP73 levels and the above indices are exhibited in Table I and Fig. 2.

Serum GP73 levels significantly increased concurrently with a reduction in ALB levels, and there was a significant difference between ALB expression level groups (all P<0.001), except the group with an ALB level of >45 g/l (Fig. 2A). A significant elevation of serum GP73 level was observed with an increase in TBIL level, and significant differences were identified between groups (Fig. 2B; all P<0.05). The serum GP73 level was significantly increased in correlation with an elevation in ALT and AST levels, and there was a significant difference between groups (Fig. 2C and D, all P<0.01), except for the group with an ALT level of <40 IU/l. Serum GP73 levels were negatively correlated with ALB levels (Fig. 2E; *r*=-0.524; P<0.0001), but positively correlated with TBIL levels (Fig. 2F; *r*=0.572; P<0.0001), ALT levels (Fig. 2G; *r*=0.498; P<0.0001) and the AST level (Fig. 2H; *r*=0.532; P<0.0001).

### Serum GP73 levels are positively correlated with serum liver fibrosis indices and liver tissue pathology

Serum markers are indirect serological markers of liver fibrosis, whereas liver biopsies represent the ‘gold standard’ for the diagnosis of liver fibrosis (19,20). In order to evaluate the correlation between serum GP73 levels and disease progression in patients with chronic HBV infections, the serum HA, CIV, LN and PIIINP levels of liver fibrosis indices were determined in 183 patients with CHB, and liver biopsy was performed on 91 patients with CHB (Table II). Serum GP73 level was found to positively correlate with serum HA (*r*=0.460), CIV (*r*=0.581), LN (*r*=0.604) and PIIINP levels (*r*=0.592) (Table II, all P<0.0001).

**Figure 3:**
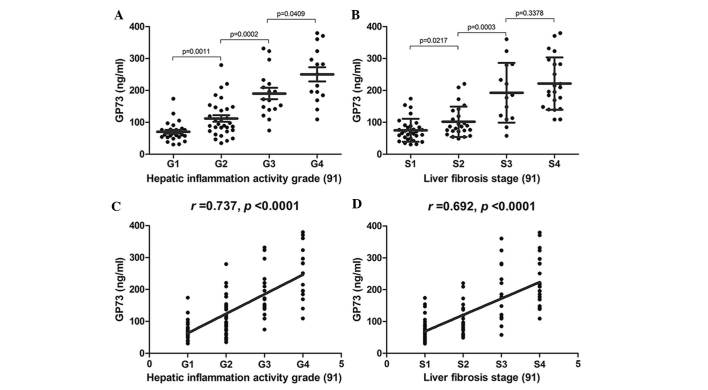
Serum GP73 level is positively correlated with the pathological grading and staging of liver tissues in chronic hepatitis B. Changes in serum GP73 levels in patients with (A) hepatic inflammation activity grades G1–G4 and (B) liver fibrosis stages S1–S4. Correlation between serum GP73 level and pathological (C) grading and (D) staging of liver tissues. GP73, Golgi protein 73.

The correlation between serum GP73 levels and liver pathological grading and staging are illustrated in Fig. 3. Serum GP73 levels were significantly increased with increased hepatic inflammation activity grade (*F*=51.50; P<0.0001) and increased liver fibrosis stage (*F*=28.85; P<0.0001). There was a significant difference amongst all hepatic inflammation activity grades (G1–G4) and amongst the liver fibrosis stages (S1–S3), regardless of the serum GP73 levels (Fig. 3A and B; all P<0.05). Serum GP73 levels were positively correlated with hepatic inflammation activity grade (Fig. 3C; *r*=0.737; P<0.0001) and liver fibrosis stage (Fig. 3D; *r*=0.692; P<0.0001).

### GP73 protein expression is correlated with hepatic inflammation grade and stage in liver tissue samples

**Figure 4:**
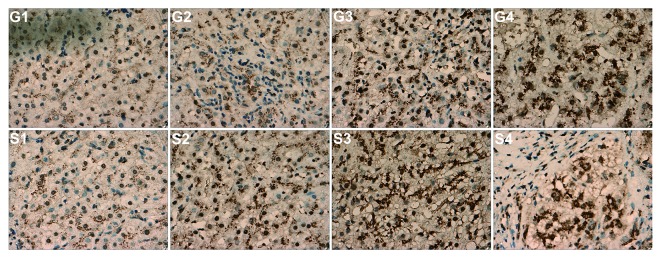
GP73 expression in pathological grading and staging of liver tissue of 16 patients with chronic hepatitis B (8 individual patients, representative of the total 16 evaluated). GP73 expression was scattered in the cytoplasm of hepatocytes, but not in the infiltrating inflammatory cells (G2 and S4) or within the fibrous septa (S4). G1 and S1, weakly positive for GP73; G2 and S2, positive for GP73; G3 and S3, moderately positive for GP73; G4 and S4, highly positive for GP73 (magnification, ×400). GP73, Golgi protein 73; G1–G4, hepatic inflammation activity grades; S1–S4, liver fibrosis stages.

GP73 protein expression was validated in liver tissue specimens of patients with chronic HBV infections by staining biopsy samples. Immunohistochemical analysis indicated that the GP73 protein was expressed in a small number of hepatocytes in normal liver tissue, whereas in patients with chronic HBV infections, GP73 expression was identified scattered in the cytoplasm of hepatocytes, but not in the infiltrating inflammatory cells or within the fibrous septa (Fig. 4, G2 and S4). In patients with mild CHB, the GP73 staining was brown and the granules were fine, and scattered in the cytoplasm of liver cells, suggesting weakly positive GP73 expression. In severe CHB, GP73 staining was brown and the granules were lumpy, and diffusely distributed in the cytoplasm of liver cells, suggesting moderately positive GP73 expression. In samples from patients with HBV-DLC, GP73 staining was dark brown and the granules coarse blocks, widely distributed in the parenchyma of the liver cytosol, suggesting strong positive expression. These results indicated that the expression levels of GP73 increased with disease progression from mild to severe CHB and HBV-DLC. Compared with mild CHB tissue specimens, GP73 was significantly expressed in severe CHB and HBV-DLC.

To further validate GP73 expression in liver tissue with regard to various hepatic inflammation grades (G0–G4) and fibrosis stages (S0–S4), GP73 protein was stained in various biopsy samples. Immunohistochemical analysis demonstrated that GP73 protein expression increased gradually with increased liver histological inflammation activity grade and liver fibrosis stage. The results of the immunohistochemical analyses are shown in Fig. 4.

### Serum GP73 levels are decreased following clinical remission of CHB

**Figure 5:**
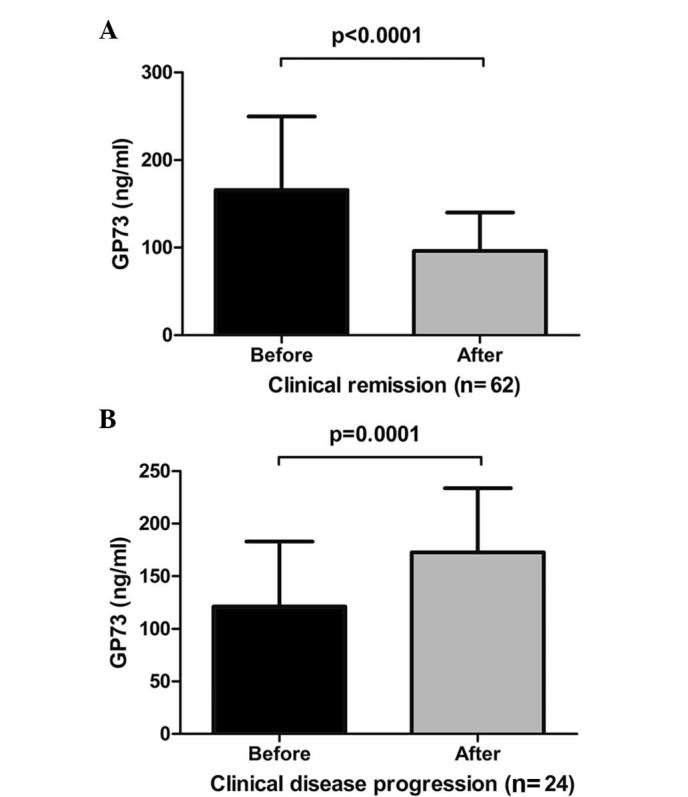
Serum GP73 level correlates with prognosis in patients with CHB. (A) Patients with CHB that reached clinical remission. (B) Patients with CHB that exhibited clinical progression. CHB, chronic hepatitis B; GP73, Golgi protein 73.

Following six months of antiviral therapy, 62 (72.09%) patients with CHB reached clinical remission with significantly decreased serum GP73 levels (Fig. 5A; *t*=6.699; P<0.0001). However, 24 (27.91%) patients with CHB that had achieved clinical progression, retained elevated levels of serum GP73 (Fig. 5B, *t*=-4.547, P=0.0001). These alterations in serum GP73 are presented in Table III.

## Discussion

GP73 is also known as a type II Golgi transmembrane protein (Golgi phosphoprotein 2, GOLPH2) and Golgi membrane protein (Golgi membrane protein 1, GOLM1), and has an estimated molecular weight of 73 kDa (5). The *GP73* gene is located on chromosome 9 and was originally cloned from a library derived from liver tissue of a patient with adult giant-cell hepatitis (5). The function and regulatory mechanisms of GP73, however, have remained elusive. Significant upregulation of GP73 has been detected in the hepatocytes of patients with hepatocellular carcinoma, and therefore GP73 may present a potential novel serum marker of hepatocellular carcinoma (21–28). The potential predictive value of GP73 in the early diagnosis of hepatocellular carcinoma has received significant attention, and elevated GP73 has been detected in patients with multiple benign liver diseases (29). Studies have demonstrated that GP73, expressed in the majority of hepatocytes, was upregulated in acute or chronic hepatitis and serum GP73 concentration was positively correlated with the progression of chronic liver diseases (8,11). Serum GP73 levels have previously been associated with liver inflammation injury and fibrosis (30,31), which is similar to the data obtained in the present study.

The correlation between serum GP73 levels and disease progression in patients with chronic HBV infections was evaluated in the present study. Serum GP73 level was elevated in patients with mild, moderate and severe CHB, patients with HBV-DLC and patients with ACLF. Serum GP73 levels were positively correlated with disease progression in patients with chronic HBV infections. Consistent with the results of the present study, a positive correlation between liver disease progression and GP73 protein expression has been previously reported, where a significant increase in GP73 was observed with the progression of liver disease (32). The correlation between serum GP73 levels and certain biochemical indices was also investigated. The results revealed that serum GP73 levels were negatively correlated with ALB levels, but positively correlated with TBIL, ALT and AST levels indicating that serum GP73 levels were associated with liver function. The higher the serum GP73 levels, the more severe the hepatic injury. This association illustrated that GP73 levels may represent a serological marker of hepatic injury. Subsequently, the correlation between serum GP73 levels and liver pathological grading and staging were further investigated. The results indicated that serum GP73 levels increased gradually with the elevation of hepatic inflammation activity grade and liver fibrosis stage. These findings demonstrated that serum GP73 levels represent an effective serological indicator of the degree of hepatic inflammation injury and the extent of liver fibrosis. The high expression of GP73 observed in acute and chronic liver diseases suggested that, whilst serum GP73 protein levels alone may not aid the elucidation of the underlying causes of liver disease, serum GP73 protein levels may be used as a serum marker for the diagnosis of liver diseases and the monitoring of liver disease progression (32).

Immunohistochemical analysis indicated that GP73 protein expression was mainly distributed in the cytoplasm of hepatocytes, and expression levels increased gradually with hepatic inflammation grade and liver fibrosis stage. The results revealed that GP73 expression was associated with disease progression in patients with chronic HBV infections, and this was consistent with liver pathological grading and staging. However, due to the limited number of immunohistochemical specimens analyzed in this study, these results require validation in a larger cohort.

In the present study, serum GP73 protein levels were positively correlated with disease progression in patients with CHB. The regulatory mechanism underlying GP73 expression is yet to be elucidated and the association between serum GP73 level and HBV replication remains unclear. A previous study indicated that HBV replication may significantly increase the expression of GP73 protein (12). A further study revealed that GP73 expression was significantly increased in liver disease due to viral causes (HBV, hepatitis C virus) compared to non-viral causes of liver disease (7). The results of these studies indicated that serum GP73 protein levels may be associated with viral infection or replication level. However, several studies have reported contrasting results. It was reported that GP73 protein expression increased significantly in patients with various liver diseases (viral and non-viral due to drug and alcohol abuse and autoimmune hepatitis) (11), and in cases of viral and non-viral liver disease, GP73 protein levels were significantly upregulated (9,33). In another study, an elevation of serum GP73 was not caused by HBV infection (13). The results of the current study demonstrated that the serum GP73 level was not significantly associated with HBV replication, regardless of serum HBeAg (positive or negative) or serum HBV DNA contents. These results differ from those of certain studies in the literature and may be associated with selected cases, and have occurred due to a variety of factors (32). Patients with chronic HBV infections frequently exhibit immune hepatic injury due to active HBV replication (34). Hepatic injury may induce the upregulation of GP73 expression in hepatocytes, or serum GP73 levels may potentially act as a novel virus-responding protein (35) associated with clinical disease progression and disease severity in patients with chronic HBV infections.

These data indicated that GP73 upregulation may be a feature of early-stage disease in patients with chronic HBV infections. In the present study, alterations in serum GP73 levels in patients with CHB were investigated following drug treatment for six months. The levels of serum GP73 were gradually decreased concurrently with the remission of CHB disease, however for cases of clinical progression, serum GP73 levels remained elevated. These results indicated that the upregulation of GP73 expression was reversible, and that serum GP73 levels were closely associated with the prognosis of patients with CHB. The present study, in accordance with previous studies (7,11) demonstrated that the upregulation of GP73 expression was reversible. There is a possibility that GP73 may be an acute reaction protein, the expression of which is triggered by hepatocyte injury and in cases of clinical remission or progression, the serum levels of GP73 are altered. These results suggested that serum GP73 levels may represent an effective index to indicate the prognosis of clinical disease.

In conclusion, serum GP73 levels are positively correlated with disease progression in patients with chronic HBV infections, and are closely correlated with liver pathological grading and staging. Therefore, serum GP73 levels may be an important indicator in the evaluation of clinical disease progression and prognosis in patients with chronic HBV infections. Whilst serum GP73 levels were not associated with HBV replication, the upregulation of GP73 expression is associated with hepatocyte injury following HBV infection. Further studies with larger sample sizes and the use of evidence-based medicine are required in order to validate the results of the current study.

## Figures and Tables

**Table I:** Serum GP73 levels associated with chronic HBV infection, liver function, serum HBeAg and HBV DNA contents.

		Serum GP73 level (ng/ml)	
		
Parameter	Patients (n)	Mean ± SD	95% CI
Clinical diagnosis	253^a^		
Mild CHB	53	74.14±36.65	64.04–84.24
Moderate CHB	80	139.78±67.05	124.86–154.70
Severe CHB	50	243.31±74.39	222.17–264.45
ACLF	35	304.45±98.48	270.62–338.28
HBV-DLC	35	255.46±92.02	223.85–287.07
ALB (g/l)	183^b^		
<35	24	236.66±80.49	202.67–270.65
35–40	61	158.92±92.16	135.31–182.52
40–45	78	104.45±57.18	91.55–117.34
>45	20	91.59±49.88	68.25–114.93
TBIL (μmol/l)	183^b^		
<34.2	139	111.14±64.83	100.27–122.02
34.2–85.5	20	192.07±82.18	153.61–230.54
>85.5	24	252.58±85.13	216.63–288.53
ALT (U/l)	183^b^		
<40	18	88.76±54.79	61.51–116.02
40–120	52	90.23±56.52	74.49–105.96
120–400	59	145.83±86.39	123.32–168.35
>400	54	193.68±83.40	170.91–216.44
AST (U/l)	183^b^		
<40	38	78.62±41.88	64.85–92.38
40–120	65	112.91±67.44	96.20–129.62
120–400	53	163.54±79.51	141.63–185.46
>400	27	235.48±87.26	200.96–269.99
HBeAg	183^b^		
Positive	117	136.76±86.21	118.32–155.63
Negative	66	139.52±81.73	121.17–165.92
HBV DNA(copies/ml)	183^b^		
<1.0E+06	29	143.52±96.15	102.31–186.52
1.0E+06 – 1.0E+07	40	151.16±92.32	105.71–191.65
1.0E+07 – 1.0E+08	61	155.21±68.16	123.29–183.51
>1.0E+08	53	136.96±81.95	98.93–179.67

A total of ^a^253 and ^b^183 cases with chronic hepatitis B. GP37, Golgi protein 37; HBV, hepatitis B virus; CHB, chronic hepatitis B; ACLF, acute-on-chronic liver failure; HBV-DLC, HBV-associated decompensated liver cirrhosis; ALB, albumin; TBIL, total bilirubin; ALT, alanine aminotransferase; AST, aspartate aminotransferase; HBeAg, hepatitis B e antigen; SD, standard deviation; CI, confidence interval.

**Table II:** Correlations between serum GP73 level, serum liver fibrosis indices and liver tissue pathology.

	Serum GP73 level (ng/ml)		
	
Parameter	Patients (n)	Mean ± SD	95% CI
Serum liver fibrosis index	183^a^		
HA	183	180.76±185.69	152.03–209.48
CIV	183	214.00±210.46	181.45–246.55
LN	183	209.49±180.17	180.17–238.82
PIIINP	183	216.55±200.09	185.60–247.50
GP73	183	168.37±95.93	153.53–183.20
Pathological grading and staging of liver tissues			
Hepatic inflammation grade	91^b^		
G1	28	70.55±29.63	59.06–82.04
G2	31	112.08±57.26	91.08–133.09
G3	17	190.27±73.19	152.64–227.90
G4	15	250.28±85.76	202.79–297.77
Liver fibrosis stage	91^b^		
S1	31	75.31±35.76	62.19–88.43
S2	24	101.92±47.67	81.79–122.04
S3	14	192.60±93.47	138.63–246.57
S4	22	221.38±83.05	184.99–257.76

A total of ^a^183 and ^b^91 cases with chronic hepatitis B undergoing pathological examinations of liver tissues. GP73, Golgi protein 73; SD, standard deviation; CI, confidence interval; HA, hyaluronic acid; CIV, type IV collagen; LN, laminin; PIIINP, type III procollagen.

**Table III:** Changes in serum golgi protein 73 expression prior to and following treatment.

Clinical prognosis	Patients (n)	Mean ± SD	95% CI
Clinical remission	62^a^		
Prior to treatment	62	165.56±84.09	144.20–186.91
Following treatment	62	96.13±43.99	84.96–107.30
Clinical progression	24^b^		
Prior to treatment	24	121.09±61.97	94.92–147.26
Following treatment	24	172.75±61.20	146.90–198.59

a Sixty-two and

b 24 cases with chronic hepatitis B.

SD, standard deviation; CI, confidence interval.
